# Development of the major trauma case review tool

**DOI:** 10.1186/s13049-017-0353-5

**Published:** 2017-02-28

**Authors:** Kate Curtis, Rebecca Mitchell, Amy McCarthy, Kellie Wilson, Connie Van, Belinda Kennedy, Gary Tall, Andrew Holland, Kim Foster, Stuart Dickinson, Henry T. Stelfox

**Affiliations:** 1 Sydney Nursing School, The University of Sydney, 88 Mallet Street, Camperdown, NSW Australia; 2 St George Clinical School, Faculty of Medicine, University of New South Wales, Gray St, Kogarah, NSW Australia; 3 Australian Institute of Health Innovation, Faculty of Medicine and Health Sciences, Macquarie University, Sydney, Australia; 4 NSW Institute of Trauma and Injury Management, Level 4, Sage Building, 67 Albert Avenue, Chatswood, NSW Australia; 5 NSW Ambulance, Level 2, Sydney Ambulance Centre, Garden St Eveleigh, NSW 2015 Australia; 6 Sydney Medical School, The University of Sydney and The Children’s Hospital at Westmead, Sydney, NSW Australia; 7 NorthWestern Mental Health & School of Nursing, Midwifery & Paramedicine, Australian Catholic University, Level 1 North, City Campus, The Royal Melbourne Hospital Grattan Street, Parkville, VIC 3050 Australia; 8 Human Risk Solutions, Victoria, Australia; 9 Departments of Critical Care Medicine, Medicine and Community Health Sciences, O’Brien Institute for Public Health, University of Calgary, Calgary, Canada

**Keywords:** Injury, Quality, Safety, Peer review, Adverse event, Mortality, Morbidity, Human factors, Organizational factors, Emergency

## Abstract

**Background:**

As many as half of all patients with major traumatic injuries do not receive the recommended care, with variance in preventable mortality reported across the globe. This variance highlights the need for a comprehensive process for monitoring and reviewing patient care, central to which is a consistent peer-review process that includes trauma system safety and human factors. There is no published, evidence-informed standardised tool that considers these factors for use in adult or paediatric trauma case peer-review. The aim of this research was to develop and validate a trauma case review tool to facilitate clinical review of paediatric trauma patient care in extracting information to facilitate monitoring, inform change and enable loop closure.

**Methods:**

Development of the trauma case review tool was multi-faceted, beginning with a review of the trauma audit tool literature. Data were extracted from the literature to inform iterative tool development using a consensus approach. Inter-rater agreement was assessed for both the pilot and finalised versions of the tool.

**Results:**

The final trauma case review tool contained ten sections, including patient factors (such as pre-existing conditions), presenting problem, a timeline of events, factors contributing to the care delivery problem (including equipment, work environment, staff action, organizational factors), positive aspects of care and the outcome of panel discussion. After refinement, the inter-rater reliability of the human factors and outcome components of the tool improved with an average 86% agreement between raters.

**Discussion:**

This research developed an evidence-informed tool for use in paediatric trauma case review that considers both system safety and human factors to facilitate clinical review of trauma patient care.

**Conclusions:**

This tool can be used to identify opportunities for improvement in trauma care and guide quality assurance activities. Validation is required in the adult population.

**Electronic supplementary material:**

The online version of this article (doi:10.1186/s13049-017-0353-5) contains supplementary material, which is available to authorized users.

## Background

As many as half of all patients with major traumatic injuries do not receive ideal care, with between 2.5% and 14% of medical errors in trauma deaths determined as potentially clinically preventable [1]. The 2014 Australian Trauma Registry report demonstrated a variance in mortality rates between states and hospitals [2], such variance is reported across clinical specialties and the globe [3, 4]. The literature also suggests that there is great variability in the quality of care for injured youth [5, 6], that deficiencies exist in the quality of care for 8% to 45% of severely injured children, and that 6% to 32% of in-hospital deaths are preventable [7]. This variance in in-hospital mortality between hospitals highlights the need for a systematic, comprehensive system for monitoring and reviewing patient care to inform processes for change to ultimately improve patient outcomes.

Trauma centres have a wide and varying range of trauma quality improvement projects, and initiatives, including morbidity and mortality meetings. Such meetings are longstanding throughout healthcare for review of patient deaths and complications, however, there remains a need to standardise the approach taken to review cases across hospitals and other trauma care providers [8]. This approach ought not to be hospital-centric and silo driven and should include elements of trauma system safety and human factors [9]. A process with standardized criteria (definitions) and measures of trauma care quality, along with consistent approaches to measurement, monitoring and reporting between hospitals is required to ascertain areas for improvements in care and identify corrective strategies. Further, the impacts of the trauma system should include all phases of care including prehospital, trauma triage criteria, hospital type and interfacility transfer, focusing on timeliness and appropriateness of care [10].

Within hospitals, there have been a range of classification frameworks and taxonomic tools developed to attempt to identify the causal factors of adverse events from a human factors perspective [11]. Many of the frameworks developed have been based on Reason’s Organisational Accident Causation Model [12], such as the London Protocol [13]. Several of these do not consider the underlying causes of adverse incidents, however, nor do they consider all human factors, including human behavior (ie. human error), that may contribute towards an adverse event or a potential adverse event occurring.

Currently, there is no published, evidence-informed standardised tool that considers both system safety and human factors for use in adult or paediatric trauma case peer review. To address this evidence-practice gap, the aim of this research was to develop and validate a trauma case review tool to facilitate clinical review of trauma patient care that addresses the recommendations of the World Health Organization (WHO) [8] and Australasian Trauma Quality Improvement Program Guidelines [2].

### Aim

To describe the development and validation of a trauma case review tool to facilitate peer-review of adverse events in paediatric major trauma designed to extract information to facilitate monitoring, inform change and facilitate loop closure.

## Methods

This trauma case review tool is intended for use to facilitate peer-review of major paediatric trauma cases flagged for analysis as a result of an adverse event and was developed during a state-wide, prospective paediatric trauma system evaluation in Australia’s most populous State, New South Wales [14]. Development was multi-faceted, beginning with a review of the literature on trauma audit tools. Data were extracted from the literature to inform iterative tool development using a consensus approach, which was then followed by pilot and inter-rater reliability testing. Each step is described below.

### Review of literature

A review of key principles from the WHO Trauma Quality Improvement Program Guidelines [8], National Safety and Quality Framework [15], the Institute of Medicine [16] and the London Trauma Protocol [17] along with the international literature on Trauma audit tools was conducted. Electronic database search was conducted using the terms “injury”, “audit”, “tool”, “peer review”.

### Development of the trauma case review tool

Extraction of data from the literature identifying categories of factors found to be causally related to adverse events was conducted and a draft tool containing seven components that considered the trauma system, and human factors was developed. A pre-existing, validated, hierarchical human factors framework was included in the tool [18], consisting of three levels to categorise the human factors contributing to any care delivery problems (Section 6).

The draft tool was reviewed by the NSW Institute of Trauma and Injury Management’s Clinical review committee and then trialed by five experienced trauma clinicians (including a trauma nurse, emergency physicians, and surgeons) using medical records from three de-identified paediatric trauma cases from different hospitals. Following feedback from the reviewers, refinements and retesting of the tool with additional de-identified cases was conducted.

### Classification of terms

Information on the role of error in any adverse events was identified for the staff action-related classifications involving medical task failures, monitoring tasks, delays, misdiagnoses, or medication issues. Error was classified using Rasmussen’s [19] skill, rule or knowledge-based error classifications, or a violation classification [20]. Skill-based errors referred to unintentional failures in the execution of a well-rehearsed action or routine task that required little conscious attention. Rule-based errors referred to unintentional failures during activities conducted in familiar situations that were controlled by stored rules. Knowledge-based errors referred to unintentional failures during a novel situation that required conscious analytic processing and stored knowledge. A violation was considered to be an intentional failure to follow accepted work practices, guidelines or procedures during the execution of a task. It is noted that within this classification system a violation does not indicate the intent to cause harm.

### Data collection

Each of the clinical reviewers was provided verbal instruction on how to use the tool and a data dictionary and each signed a confidentiality agreement. Clarification on definitions and aspects of the tool was provided as required, and modifications made accordingly following the testing.

For the pilot, the reviewers were emailed links to the de-identified files using the secure Cloudstor platform (Australia Academic and Research Network). A second round of testing following refinement of the tool was conducted by eight trauma clinicians who used the tool to classify eight de-identified paediatric trauma cases. This group included the same clinicians who reviewed the pilot tool, as well as interstate and international clinicians from nursing, surgical, emergency and retrieval backgrounds. For the second round of testing, a day long face-to-face meeting was held, orientation to the tool conducted, and hard copies of de-identified trauma cases provided for peer-review. Each reviewer completed the case review independently using the tool. Cases used for testing had been identified as having adverse events by the site trauma service and the age range of the injured children was 8 weeks to 15 years.

### Data management and analysis

Data from both rounds of testing were entered in ExcelTM. Double data entry was conducted to ensure accuracy. Inter-rater reliability of the adverse event causal factors and outcomes components sections of the tool was assessed using percent agreement [18, 21]. Entries had to agree exactly to be considered the same, and only those answered as ‘yes/no/not applicable’ were assessed. The percentage agreement was calculated as the ratio of the total number of ‘same’ responses for each data element divided by the total number of data elements assessed. For measurement of concordance in the human factors framework section that consists of three levels to categorise the human factors contributing to any care delivery problems, agreement pertained to raters recording the ‘same’ response for up to three levels for each factor, for example, a Level 1 factor was *‘Work Environment* - *Did the work environment contribute to difficulties in delivering the required care?”*. Level 2 of ‘Work Environment’ is the subsection within Level 1, with a possible response being *‘Light’* and a Level 3 response could have been *‘No or too little light’*.

## Results

### Review of literature

Several papers reported that they conducted a peer-review process and classified outcomes as either preventable or non-preventable [22–24]. No original research studies of a validated peer-review tool for use in trauma care were identified.

### Iterative refinement

The usability testing and pilot of the trauma case review tool by the clinical reviewers identified that several modifications of the tool needed to be made. Modifications made are presented in Table 1.

**Table 1:** Modifications made to the case peer review tool

-Addition of a timeline displaying key events in chronological order to provide a snapshot of what happened
-Addition of Section 8 which allows for the recording of positive aspects of care
-Addition of Section 9 to identify whether reviewers have had prior knowledge of the case which may impact on their review
-Addition of answer options in cases where not all options are covered
-Minor modification to the wording of some questions to avoid ambiguity
-Minor modification to the layout and structure to improve usability
-Prompts to interview relevant staff to gather further information

### Inter-rater reliability

For the pilot, the interrater reliability of the human factors and outcome components of the tool had an average 81% agreement between raters at level 1, 69% at level 2 and 69% at level 3. After refinement of the tool, the interrater reliability of the human factors and outcome components of the tool improved and had an average 86% agreement between raters at Level 1. There was a moderate decrease in reliability with 67% at level 2 and 63% at level 3.

### Final trauma case review tool

The final tool contained ten sections, including patient factors (such as pre-existing conditions), presenting problem, a timeline of events, specific services involved in the care delivery, factors contributing to the care delivery problem (including equipment, work environment, staff action, organizational factors), patient outcome, positive aspects of care and the outcome of panel discussion (Additional files 1 and 2). Each component of the tool was informed by the aforementioned literature search and is outlined in Table 2.

**Table 2:** Components of the major trauma case peer review tool

Basic information	
Record ID	This is the unique record used by the study team to identify each record
Reviewer ID	Each reviewer has a unique identification number
Date of review	For recording when the review was conducted
Date and time of injury	Key time variables allow for the development of a chronology
Age and gender	Age and gender to allow comparative analysis across groupings and determination of specific areas for education/change within the trauma system that considers age related physiology, age specific injury patterns [36, 37]
Date and time of incident(s)	Key time variables allow for the development of a chronology
Section 1: Patient factors	
Background	Such as whether the child is Aboriginal or Torres Strait Islander, culturally and linguistically diverse or a refugee to assist with the identification of potentially vulnerable groups and engagement with appropriate stakeholders when required
Previous location and source of referral	Primary presentation, secondary presentation (e.g. inter-hospital transfer) and source of referral (e.g. self, road ambulance) to assist with mapping of patient flow and identification of potential areas of deficits
Other patient factors	This component attempts to capture the unique characteristics of the patient in the context of their presentation including: complexity and acuity of presentation; behavioural and social factors
Section 2: Presenting problem/diagnosis	
Injury mechanism, injuries, and signs and symptoms on presentation	These sections capture the cause and nature of the injury
Section 3: Timeline of events	
Timeline of events	Timeline of events in chronological order
Section 4: General incident information	
Did the patient die?	To determine whether the child died as a result of their injuries and to assist with further questioning
Phase of care did the patient died in (pre-hospital/during transport/in-hospital/which ward?)	To provide a construct on where the incident occurred, allowing monitoring of one point of care or service
Was a toxicology screen/post mortem conducted? If yes, what type was completed and is the report available?	Autopsy reports are a valuable source of information and provide an important adjunct to any investigation of factors potentially contributing to patient mortality [8]
Category of the problem (either clinical, systems or communication)	To assist with the determination of how the clinical deficit occurred and to allow comparative analysis across groupings and determination of specific areas for education/change within the trauma system [27]
Section 5: Specific services involved in the care delivery problem	
Specific department and staff involved in the care delivery problem	This multiple choice and free text response section allows for determination of services involved in the care delivery problem
Section 6: Factors contributing to the care delivery problem	
Equipment	Including: lack of medical equipment, medical equipment breakage or failure, equipment failure (design), medical equipment not elsewhere classified, non-medical equipment and medical supplies
Work environment	Including: light, temperature, noise, physical layout, security and work environment not elsewhere classified
Staff action	Including: verbal communication and written documentation issues, medical task failure, monitoring, delay, misdiagnosis, medication issue and human factors not elsewhere classified
Patient	Including: physical health, health state, communication issues, medication, toxicology, clothing, and patient characteristics not elsewhere classified
Organisational factors	Including: work practices, policies or guidelines, supervision, organisational resources, work pressure and organisational factors not elsewhere classified
Individual factors	Including: training, experience, fatigue, stress and individual factors not elsewhere classified
Other factors	This is a free text response for factors the reviewer feels are not addressed in the previous categories
Section 7: Outcome	
Best description of the incident	How the incident can be best described ranging from clinically preventable to clinically non-preventable death, near miss of death, near miss of incident that did not result in death, preventable error causing lasting disability or no problems identified
Section 8: Positives of care	
Positive aspects of care the patient received	This free text response allow for the recording of positives of care the patient received
Section 9: Prior knowledge	
Reviewer prior knowledge of the case	Included to identify whether the reviewer have had prior knowledge of the case which may affect their review of the case
Section 10: Panel discussion	
Summary of review and recommendations	Free text response to allow for a summary of the review and recommendation for corrective strategies after panel discussion
Interview of staff involved?	To allow staff details to be recorded if staff are recommended for interview to obtain further information for completing the assessment

### Trauma system components

The first sections (Sections 1–3) of the case review tool include demographic and injury information to allow for the development of a chronology and consideration of age- and any patient- specific physiology [25]. The following sections (Sections 4–5) collect clinical management and service delivery information in the standardised Airway, Breathing, Circulation format known to improve trauma patient outcomes when followed [26]. This clinical categorisation of treatment in common trauma language facilitates case-specific areas of improvement and longer term monitoring of areas of care that may require widespread education or intervention for change [1, 23, 27, 28]. It also enables identification of compliance with specific local or statewide trauma management guidelines or protocols. Detailed information on the locations and services involved in a particular incident or near miss will also allow specific intervention if required, and ongoing monitoring to provide evidence for change.

### Consideration of causal factors

Section six records information on human factors that may have influenced clinical practice including: equipment, work environment, staff action, patient, organizational, individual and other factors. The human factors component was adapted from the Human Factors Classification Framework for patient safety [18]. This framework was adopted as previous inter-rater reliability for human factors classifications has been demonstrated to be high [29] and the approach was based on James Reason’s model of organisational incidents [20].

**Fig. 1:**
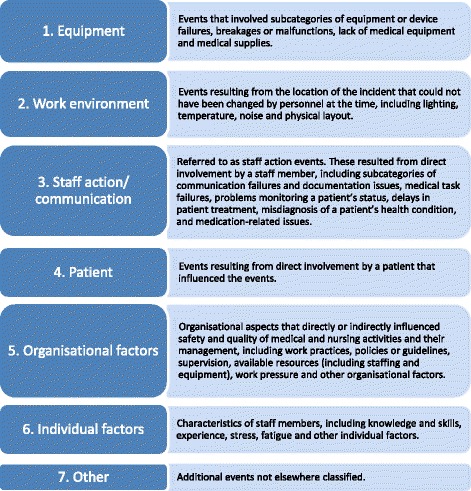
Factors contributing to the care delivery problem

The human factors component was used to identify influencing or causal factors that were thought to play a role in leading to the adverse event. Each causal factor was classified into one of seven categories (Fig. 1) then a number of subcategories.

## Discussion

This manuscript describes the development, refinement and reliability of a trauma case review tool to facilitate peer-review of adverse events in pre- and in-hospital care provision for major trauma patients. The major trauma case review tool, informed by evidence, is designed to extract trauma system safety and human factor causal information to facilitate monitoring, inform change and facilitate loop closure in the provision of care of major trauma patients.

This tool incorporates human factors with the intent of enhancing current Morbidity and Mortality review practices. Following an adverse event it is common across industries for the focus to be understanding what happened. The focus in a human factors approach is to understand why an adverse event occurred. Identifying the underlying systemic factors that contributed to the occurrence of an adverse event and the event outcomes will assist in understanding why particular decisions and actions occurred. It can also provide insight as to what can be done to prevent these events from occurring in the future, by addressing the underlying causal factors. Incorporating a human factors review component within this case review tool seeks to place greater focus on why the event occurred. This involves investigating human, equipment, organisational and environmental factors that may have contributed to the occurrence of the event which is in line with WHO recommendations that the review process should identify clinical errors, consider work system factors that contributed to the occurrence of the errors, and facilitate corrective action plans [8].

Adverse events are directly linked to actual harm resulting from (or omission of) health care and are generally independent of the patient’s disease. An adverse event may occur despite the correct care being given under correct circumstances but ultimately associated with a poor outcome [30]. Errors are one aspect of failure in the processes of care. While historically there has been a focus on the individuals that perpetrated the error we know that human performance and the occurrence of errors is influenced by a range of factors in the environment and organisational systems. It is through understanding these influences that we can identify potential improvements in the system and thereby reduce the re-occurrence of similar incidents in the future. The approach of applying a structured framework of these influencing/causal factors is particularly suited to the case review methodology where the detailed data around individual action may not be available but environmental and organisational factors can be identified by experienced reviewers. This tool records adverse events, but also informs understanding of the areas of the system that require further investigation, and monitoring for trends to highlight where change is needed.

Adverse patient events require monitoring, and, for system-wide change, active monitoring and investigation of local, state-based and a national registry(s) is optimal as they are designed to provide information that can be used to improve the efficiency and quality of trauma care. However, trauma registries require more rigour to be reliable in the quality of the reproducible data [31, 32], and they lack the detail afforded by a human factors enhanced peer-review process. There are growing efforts to improve patient safety in trauma and quantification of the burden of iatrogenic harm could catalyse awareness and stimulate changes in trauma practice and healthcare policy [33]. Future work should include integration of the two.

There are some limitations to the development of the tool. Evaluation of the trauma case review tool in this study was restricted to the paediatric trauma population whose anatomical, physiological and psychological management varies significantly compared to adults [34, 35]. There was a wide range of ages of children examined using the development of the tool, and recognition of these differences in a review tool, including age-specific injury patterns and appropriate care of children’s families should be included [36, 37]. Future clinical care review tools should also include the patient experience where possible, although challenges remain in how best to obtain this information. Doyle et al. [38] found a positive association between patient experience and measures of the technical quality of care and adverse events and support the inclusion of patient experience as one of the central pillars of quality in healthcare.

This tool was tested using a retrospective review of medical records. This method provided informative timelines and information about procedures and immediate patient outcomes. However it was more difficult to extract human factors information. The tool is recommended to be used in conjunction with staff who were part of the treating team, so they can be involved in the review process to ensure accurate and informed classification of human factors. It is anticipated that more intimate knowledge of situation and organizational factors will allow even more useful information to be captured in the human factors section of the tool. To attempt to link the adverse event to longer term patient outcomes would require linkage with a trauma registry that collects such information or a follow-up study with adversely affected patients.

The next stages of evaluation of the trauma case review tool could include a trial of the tool with an adult population and heuristic evaluation (that is, usability evaluation by a human factors expert against a set of usability rules/principles) and could entail observing clinicians using the tool. This would include consideration of the clinicians’ ability to understand and apply the human factors component of the tool, which requires a degree of understanding of human factors principles. Also, although the case review tool is evidence-informed, and has been piloted by trauma clinicians it requires validation for sensitivity and specificity in identifying causes of adverse events. A retrospective cohort study to measure operating characteristics and prospective implementation of the tool into quality assurance activities to gauge how it is received, how well it identifies adverse events, and what type of quality improvement activities it spurs, would be valuable. Such evaluation could also lead to the development of trauma specific trigger tools, to be used in real time as a predictor for adverse events and provide the basis of a measurable trauma quality improvement program [33, 39].

## Conclusions

As many as half of all patients with major traumatic injuries do not receive the recommended care and up to 14% of medical errors in trauma deaths are potentially preventable. This research has developed an evidence-informed tool for use in trauma case review that considers system safety and human factors to facilitate clinical review of trauma patient care. This tool can be used to identify opportunities for improvement in trauma care and guide quality assurance activities.

## Additional files

Additional file 1: Major trauma case peer review tool. (PDF 422 kb)
Additional file 2: Data dictionary. (PDF 289 kb)

## Abbreviations

WHO: World Health Organization.
