# Supplementary material for: Development of the major trauma case review tool
Source: Scand J Trauma Resusc Emerg Med. 2017 Feb 28;25:20. doi: 10.1186/s13049-017-0353-5 (PMC5330157; doi:10.1186/s13049-017-0353-5)
Supplement: Additional file 2: — Data dictionary. (PDF 289 kb) [file 13049_2017_353_MOESM2_ESM.pdf]

MAJOR TRAUMA CASE PEER REVIEW TOOL

Data Dictionary

July 2016

List of Data Variables

Basic information 5
- Variable: Record ID 5
- Variable: Reviewer ID 5
- Variable: Date of review 5
- Variable: Date of injury 5
- Variable: Time of injury 6
- Variable: Age 6
- Variable: Gender 6
- Variable: Date and time of incident(s) 6

Section 1: Patient factors 7
- Variable: Background 7
- Variable: Previous location 7
- Variable: Other patient factors 7
- Variable: Source of referral 8

Section 2: Presenting problem/diagnosis 9
- Variable: Injury mechanism 9
- Variable: Injuries 9
- Variable: Signs and symptoms on presentation 9

Section 3: Timeline of events 10
- Variable: Timeline 10

Section 4: General incident information 11
- Variable: Did the patient die? 11
- Variable: If yes, did the patient die pre-hospital? 11
- Variable: If yes, did the patient die during transport? 11
- Variable: Did the patient die in-hospital? 11
- Variable: If yes, in which location did the patient die? 12
- Variable: Was a toxicology screen conducted? 12
- Variable: Was a post mortem conducted? 12
- Variable: If yes, what type of post mortem was completed? 13
- Variable: Is the report available? 13
- Variable: Categorise the problem 13

Section 5: Specific services involved in the care delivery problem 15

Variable: Department 15
Variable: Specialty level of staff 15
Section 6: Factors contributing to the care delivery problem 16
Variable: Did any of the following factors contribute to difficulties in delivering the required care? 16
Section 7: Outcome 19
Variable: Tick the option that best describes the incident 19
Section 8: Positives of care 20
Variable: Positives of care 20
Section 9: Prior knowledge 21
Variable: Did you have prior knowledge of this case? 21
Section 10: Panel discussion 22
Variable: Summary of review and recommendations 22
Variable: Interview staff involved? 22

Basic information

Variable: Record ID
Variable name: ID
Definition: A consecutive number allocated to each record
Format: 2 digit numeric
Coding frame: 01 Record 1
02 Record 2
03 Record 3
04 Record 4
Comments: This is the unique record used by the study team to identify each record
Guideline for use: Enter numeric code

Variable: Reviewer ID
Variable name: reviewer_ID
Definition: A number allocated to identify each reviewer
Format: 2 digit numeric
Coding frame: 01 Reviewer 1
02 Reviewer 2
03 Reviewer 3
04 Reviewer 4
Comments: Each reviewer has a unique identification number
Guideline for use: Enter numeric code

Variable: Date of review
Variable name: date_reviewed
Definition: Date the review of the record is completed
Format: 6 digit numeric
Coding frame:
Comments:
Guideline for use: Enter date as DD/MM/YY

Variable: Date of injury
Variable name: date_injury
Definition: Date the child was injured
Format: 6 digit numeric
Coding frame:
Comments: Key time variables allow for the development of a chronology
Guideline for use: Enter date as DD/MM/YY

Variable: Time of injury

Variable name: time_injury
Definition: Time the child was injured
Format: 4 digit numeric
Coding frame:
Comments: Key time variables allow for the development of a chronology
Guideline for use: Enter the time in 24 hour format eg. 1830

Variable: Age

Variable name: age
Definition: Age of the child to whom the incident occurred in years and months
Format: 3 digit numeric
Coding frame:
Comments: To allow comparative analysis across groupings and determination of specific areas for education/change within the trauma system that considers age related physiology, age specific injury patterns
Guideline for use: Enter the child's age in years and months

Variable: Gender

Variable name: gender
Definition: Gender of the child to whom the incident occurred
Format: 1 digit numeric
Coding frame: 1 Male
2 Female
9 Not recorded
Comments:
Guideline for use: Select male or female

Variable: Date and time of incident(s)

Variable name: date
Definition: Date and time of the incident(s) that occurred
Format: Text
Coding frame:
Comments: Key time variables allow for the development of a chronology
Guideline for use: Provide a brief free text description of the incident(s) that occurred and the approximate date (DD/MM/YY) and time (24 hour format) when it occurred

---

Section 1: Patient factors

Variable: Background

Variable name: background
Definition: Do any of the following apply to the child: identifies as Aboriginal or Torres Strait Islander, is from a culturally and linguistically diverse background, is a refugee?
Format: 1 digit numeric
Coding frame: 1 Aboriginal or Torres Strait Islander
2 Culturally and linguistically diverse (please specify)
3 Refugee
Comments: To assist with the identification of potentially vulnerable groups and engagement with appropriate stakeholders when required
Guideline for use: Select all options that apply to the child. If 'culturally and linguistically diverse' is selected, please specify language spoken at home

----

Variable: Previous location

Variable name: prev_location
Definition: Was the presentation a primary, secondary or other type of presentation?
Format: 1 digit numeric
Coding frame: 1 Primary presentation
2 Secondary presentation (inter-hospital transfer)
3 Other (please specify)
Comments: To assist with mapping of patient flow and identification of potential areas of deficits
Guideline for use: Select the option that applies to the child. If 'other' is selected, please provide a brief free text description

----

Variable: Other patient factors

Variable name: other_factors
Definition: Was the child in an altered mental state? Is the child obese, known to have a developmental delay or co-morbidities?
Format: 2 digit numeric
Coding frame: 1 Altered mentation
11 Alcohol intoxication
12 Substance misuse
13 Mental disturbance
2 Obese
3 Known developmental delay
4 Co-morbidities (please specify)
Comments: To capture the unique characteristics of the patient in the context of their presentation
Guideline for use: Select all options that apply to the child. If 'co-morbidities' is selected, please specify co-morbidities

----

Variable: Source of referral

Variable name: referral_source
Definition: Source where notification of the injured child came from
Format: 2 digit numeric
Coding frame: 1 Self
2 Relative
3 Carer
4 General practitioner (GP)
5 Road ambulance (paramedic)
6 Adult retrieval service (road/helicopter/fixed wing)
7 Paediatric medical retrieval service (road/helicopter/fixed wing)
8 Other
Comments: To assist with mapping of patient flow and identification of potential areas of deficits
Guideline for use: Select the option that applies to the child. If ‘other’ is selected, please provide a brief free text description

---

Section 2: Presenting problem/diagnosis

Variable: Injury mechanism
Variable name: injury_mechanism
Definition: Event or circumstance associated with the cause of injury
Format: Text
Coding frame:
Comments:
Guideline for use: Provide a brief free text description of the injury mechanism, to provide information on specific circumstances of injury. For example:
- fall from a balcony while climbing the rail
- MVC rollover, father driving, child unrestrained
- travelling on holidays with family, had fall at playground
- child playing with brother on lounge and fell through flyscreen landing on concrete

---

Variable: Injuries
Variable name: injuries
Definition: Nature of the injuries responsible for occasioning the attendance of the child to hospital
Format: Text
Coding frame:
Comments:
Guideline for use: Provide a brief free text description of the child's injuries. For example:
- Spinal cord injury
- Traumatic brain injury
- Eye injury
- Fractured femur

Variable: Signs and symptoms on presentation
Variable name: signs_symptoms
Definition: Signs and symptoms the child presented to hospital with
Format: Text
Coding frame:
Comments:
Guideline for use: Provide a brief free text description of the signs and symptoms the child presented with. For example:
- vomiting
- bleeding
- swelling
- bruising

Section 3: Timeline of events

Variable: Timeline

Variable name: Timeline
Definition: A timeline of events relevant to the incident in chronological order
Format: Text
Coding frame:
Comments: This section is pre-filled for the convenience of the reviewer
Guideline for use:

Section 4: General incident information

Variable: Did the patient die?
Variable name: die
Definition: Did the child die?
Format: 1 digit numeric
Coding frame: 1 Yes
2 No
9 Not recorded
Comments:
Guideline for use: Select 'yes' or 'no'

Variable: If yes, did the patient die pre-hospital?
Variable name: die_prehosp
Definition: Did the child die prior to reaching hospital?
Format: 1 digit numeric
Coding frame: 1 Yes
2 No
9 Not recorded
Comments: To provide a construct on where the incident occurred, allowing monitoring of one point of care or service
Guideline for use: This question is only to be answered if the child died. Select 'yes' or 'no'

Variable: If yes, did the patient die during transport?
Variable name: die_transport
Definition: Did the child die whilst being transported to hospitals?
Format: 1 digit numeric
Coding frame: 1 Yes
2 No
9 Not recorded
Comments: To provide a construct on where the incident occurred, allowing monitoring of one point of care or service
Guideline for use: This question is only to be answered if the child died pre-hospital. Select 'yes' or 'no'. If 'yes' is selected please specify who/which organisation transported the child to hospital

----

Variable: Did the patient die in-hospital?
Variable name: die_inhosp
Definition: Did the child die while being treated in hospital?
Format: 1 digit numeric
Coding frame: 1 Yes
2 No
9 Not recorded

Comments: To provide a construct on where the incident occurred, allowing monitoring of one point of care or service
Guideline for use: This question is only to be answered if the child died. Select 'yes' or 'no'

Variable: If yes, in which location did the patient die?

Variable name: death_location
Definition: Which hospital department/ward was the child located in when they died?
Format: 1 digit numeric
Coding frame: 1 ED
2 Radiology
3 Angiography
4 OT
5 ICU
6 HDU
7 Ward
8 Other (please specify)
9 Not recorded
Comments: To provide a construct on where the incident occurred, allowing monitoring of one point of care or service
Guideline for use: This question is only to be answered if the child died in-hospital. Select the most appropriate location to describe where the child died. If 'other' is selected please provide a brief free text description

---

Variable: Was a toxicology screen conducted?

Variable name: toxicology
Definition: Was forensic toxicology testing performed on the child?
Format: 1 digit numeric
Coding frame: 1 Yes
2 No
9 Not recorded
Comments: Autopsy reports are a valuable source of information and provide an important adjunct to any investigation of factors potentially contributing to patient mortality
Guideline for use: This question is only to be answered if the child died. Select 'yes' or 'no'

Variable: Was a post mortem conducted?

Variable name: post_mortem
Definition: Was a post mortem conducted to determine cause of death?
Format: 1 digit numeric
Coding frame: 1 Yes
2 No
9 Not recorded

Comments: Autopsy reports are a valuable source of information and provide an important adjunct to any investigation of factors potentially contributing to patient mortality
Guideline for use: This question is only to be answered if the child died. Select 'yes' or 'no'

Variable: If yes, what type of post mortem was completed?
Variable name: type_post_mortem
Definition: Was an open (external and internal examination of the body) or closed (external examination of the body only) post mortem conducted?
Format: 1 digit numeric
Coding frame: 1 Open
2 Closed
9 Not recorded
Comments: Autopsy reports are a valuable source of information and provide an important adjunct to any investigation of factors potentially contributing to patient mortality
Guideline for use: This question is only to be answered if a post mortem was conducted. Select 'open' or 'closed'

Variable: Is the report available?
Variable name: post_mortem_report
Definition: Is the post mortem report available?
Format: 1 digit numeric
Coding frame: 1 Yes
2 No
9 Not recorded
Comments: Autopsy reports are a valuable source of information and provide an important adjunct to any investigation of factors potentially contributing to patient mortality
Guideline for use: This question is only to be answered if a post mortem was conducted. Select 'yes' or 'no'

Variable: Categorise the problem
Variable name: problem_category
Definition: Can the problem be categorised as a clinical, systems or communication problem?
Format: 2 digit numeric
Coding frame: 1 Clinical
11 Airway (please explain)
12 Breathing (please explain)
13 Circulation (please explain)
14 Disability (please explain)
15 Exposure/temperature (please explain)
2 Systems
3 Communication

4 Unable to categorise
5 No problem identified

Comments: To assist with the determination of how the clinical deficit occurred and to allow comparative analysis across groupings and determination of specific areas for education/change within the trauma system

Guideline for use: Select the most appropriate option to describe the problem. If the problem is a clinical problem, indicate if it is related to airway, breathing, circulation, disability or exposure/temperature and provide a brief free text description

---

Section 5: Specific services involved in the care delivery problem

----

Variable: Department

Variable name: department_involved
Definition: Which departments were involved in the care delivery problem?
Format: 2 digit numeric
Coding frame: 1 Angiography
2 Cardiothoracic surgery
3 Emergency Department
4 General surgical
    5 Paediatric
    6 Non-paediatric
7 ICU/HDU
8 Mental health service
9 Neurosurgery
10 Operating theatre
11 Orthopeadic
12 Out of hospital service
    13 Fixed wing
    14 Helicopter
    15 Road
16 Pathology
17 Plastic surgery
18 Radiology
19 Rehabilitation
20 Urology
21 Other (please specify)
Comments:
Guideline for use: Select all departments involved in the incident. If ‘ambulance’ is selected please indicate if it is a ‘fixed wing’, ‘helicopter’ or ‘road’ ambulance. If ‘other’ is selected please provide a brief free text description

----

Variable: Agency/site/specialty level of staff

Variable name: staff_involved
Definition: Agency/site/specialty level of staff involved in the care delivery problem
Format: Text
Coding frame:
Comments:
Guideline for use: List the agency/site/specialty level of staff involved in the care delivery problem. For example:
- staff specialist
- P1 paramedic

Section 6: Factors contributing to the care delivery problem

Variable: Did any of the following factors contribute to difficulties in delivering the required care?

Variable name: contributing_factors
Definition: What are the contributing factors identified that led to difficulties in delivering the required care?
Format: 3 digit numeric
Coding frame: 1 Equipment

- 11 Lack of medical equipment
- 12 Medical equipment breakage or failure
- 13 Equipment failure – design
- 14 Medical equipment not elsewhere classified
- 15 Non-medical equipment
- 16 Medical supplies

2 Work environment

- 21 Light
  - 211 No or too little light
  - 212 Too much light
  - 213 Light not elsewhere classified
- 22 Temperature
  - 221 Too hot
  - 222 Too cold
  - 223 Temperature not elsewhere classified
- 23 Noise
  - 231 Too noisy
  - 232 Too quiet
  - 233 Noise not elsewhere classified
- 24 Physical layout
  - 241 Isolation
  - 242 Poor access
  - 243 Physical layout not elsewhere classified
- 25 Security
- 26 Work environment

3 Staff action

- 31 Verbal communication and written documentation issues
  - 311 Verbal communication with patient (please describe)
  - 312 Verbal communication with staff – handover (describe)
  - 313 Verbal communication with staff – not during handover
  - 314 Written communication with patient (please describe)
  - 315 Written communication with staff (please describe)
  - 316 Other communication issue (please describe)
- 32 Medical task failure
  - 321 Rule-based error
  - 323 Knowledge-based error
  - 324 Violation
  - 329 Not known
- 33 Monitoring
  - 331 Skill-based error
  - 332 Rule-based error

- 333 Knowledge-based error
- 334 Violation
- 339 Not known
- 34 Delay
  - 341 Skill-based error
  - 342 Rule-based error
  - 343 Knowledge-based error
  - 344 Violation
  - 349 Not known
- 35 Misdiagnosis
  - 351 Skill-based error
  - 352 Rule-based error
  - 353 Knowledge-based error
  - 354 Violation
  - 359 Not known
- 36 Medication issue
  - 361 Skill-based error
  - 362 Rule-based error
  - 363 Knowledge-based error
  - 364 Violation
  - 369 Not known
- 37 Human factors not elsewhere classified. Please specify:
  - 371 Skill-based error
  - 372 Rule-based error
  - 373 Knowledge-based error
  - 374 Violation
  - 379 Not known

4 Patient
- 41 Physical health (pre-existing)
  - 411 Pre-existing disease or physical disability
  - 412 Physical characteristic
  - 413 Intellectual disability
  - 414 Psychological disturbance
  - 415 Sensory impairment – vision
  - 416 Sensory impairment – hearing
  - 417 Physical health not elsewhere classified. Specify:
- 42 Health state
  - 421 Airway obstruction
  - 422 Respiratory failure
  - 423 Coagulopathy
  - 424 Uncontrolled bleeding
  - 425 Deterioration in physical condition
  - 426 Severity of injury
  - 427 Infection
  - 428 Fatigue
  - 429 Health state not elsewhere classified. Please specify:
- 43 Communication issues
  - 431 Language barrier – child
  - 432 Language barrier – parent
  - 433 Not disclosing information – unintentional
  - 434 Not disclosing information – intentional
- 44 Medication

441 Prescribed drug(s) – excessive or inappropriate use
442 Prescribed drug(s) – appropriate use
45 Toxicology
451 Alcohol consumption
452 Non-prescribed or illicit drug consumption
453 Other toxicology. Please specify:
46 Clothing
461 Flammable
462 No helmet
463 No protective wear
47 Patient characteristics not elsewhere classified
5 Organisational factors
51 Work practices, policies or guidelines
511 Work practice, but no policy/guidelines
512 Policy/guidelines exist, but are unclear/inconsistent/inadequate
513 Policy/guidelines exist, but are not followed
514 Policy/guidelines not elsewhere classified. Specify:
52 Supervision
53 Organisational resources
531 Staffing issues (eg. skill mix)
532 No bed available (at treating site)
533 No bed available (at referring site)
534 Organisational resources not elsewhere classified
54 Work pressure
55 Organisation factors not elsewhere classified. Please specify:
6 Individual factors
61 Training
62 Experience
63 Fatigue
64 Stress
65 Individual factors not elsewhere classified Please specify:
70 Other factors. Please specify:

Comments: Contributing factors are largely pre-existing conditions and include any further factor which makes a direct contribution to the occurrence of the incident. Experience refers to 'lack of skill or competence to perform task'.

Guideline for use: Select all factors that led to the incident occurring. For factors 3.1.1-3.3.6, 3.7.1-3.7.9, 4.1.7, 4.2.9, 4.5.3, 4.7, 5.1.4, 5.3.4, 5.5, 6.7 and 7 please provide a brief free text description

----

Section 7: Outcome

Variable: Tick the option that best describes the incident

Variable name: describe_incident
Definition: Was the incident preventable/near-preventable/non-preventable, was it a near miss and did it result in death/disability?
Format: 1 digit numeric
Coding frame: 1 Clinically preventable trauma death
2 Clinically near-preventable trauma death
3 Clinically non-preventable trauma death
4 Near miss of death
5 Near miss of incident that did not result in death
6 Preventable error causing lasting disability
7 No problems identified
Comments:
Guideline for use: Select the option that best describes the incident

----

Section 8: Positives of care

Variable: Positives of care

Variable name: positive_care
Definition: What were the positive aspects of care the patient received?
Format: Text
Coding frame:
Comments:
Guideline for use: Provide a brief free text description of the positive aspects of care the patient received

---

Section 9: Prior knowledge

Variable: Did you have prior knowledge of this case?

Variable name: prior_knowledge
Definition: Did the reviewer have prior knowledge of the case?
Format: 1 digit numeric
Coding frame: 1 Yes
2 No
Comments:
Guideline for use: Select ‘yes’ or ‘no’ and provide details where necessary

----

Section 10: Panel discussion

Variable: Summary of review and recommendations

Variable name: recommendation
Definition: Summary of the conclusions and recommendations of the review
Format: Text
Coding frame:
Comments:
Guideline for use: Provide a brief free text description

Variable: Interview staff involved?

Variable name: staff_interview
Definition: Should staff involved in the care delivery problem be interviewed?
Format: 1 digit numeric
Coding frame: 1 Yes
2 No
Comments:
Guideline for use: Select ‘yes’ or ‘no’ and provide details where necessary

----
