# Supplementary material for: Development of the major trauma case review tool
Source: Scand J Trauma Resusc Emerg Med. 2017 Feb 28;25:20. doi: 10.1186/s13049-017-0353-5 (PMC5330157; doi:10.1186/s13049-017-0353-5)
Supplement: Additional file 1: — Major trauma case peer review tool. (PDF 422 kb) [file 13049_2017_353_MOESM1_ESM.pdf]

Record ID ___________ Reviewer ID__________

# MAJOR TRAUMA CASE PEER REVIEW TOOL

| | |
|---|---|
| **Date of review** | __ / __ /__ |
| **Date of injury** | __ / __ /__ |
| **Time of Injury** | ____ hours |
| **Age** | _______ years _______months |
| **Gender** | ☐ Male<br>☐ Female |

| **Date and time of incident(s)** (approximate date and time any failings occurred) |
|---|
| Click here to enter text. |

# 1. Patient factors

Tick all relevant options

| | |
|---|---|
| **Background** | ☐ Aboriginal or Torres Strait Islander<br>☐ Culturally and linguistically diverse (please specify) ____________________________<br>☐ Refugee |
| **Previous location** | ☐ Primary presentation<br>☐ Secondary presentation (inter-hospital transfer)<br>☐ Other (please specify) ____________________________________________________ |
| **Other patient factors** | ☐ Altered mentation<br>  ☐ Alcohol Intoxication<br>  ☐ Substance misuse<br>  ☐ Mental disturbance<br>☐ Obese<br>☐ Known developmental delay<br>☐ Co-morbidities (please specify) ____________________________________________ |
| **Source of referral** | ☐ Self<br>☐ Relative<br>☐ Carer<br>☐ General Practitioner (GP)<br>☐ Road ambulance (paramedic)<br>☐ Adult retrieval service (road/helicopter/fixed wing)<br>☐ Paediatric medical retrieval service (road/helicopter/fixed wing)<br>☐ Other (please specify) _______________________________________________ |

# 2. Presenting problem/diagnosis

| |
|---|
| **Injury mechanism (eg. fall from swing, rear seat passenger in vehicle crash)**<br>Click here to enter text. |
| **Injuries (eg. paraplegia, traumatic brain injury)**<br>Click here to enter text. |
| **Signs and symptoms on presentation (eg. vomiting, bleeding)**<br>Click here to enter text. |

# 3. Timeline of events

Click here to enter text.

# 4. General incident information

**1. Did the patient die?**

☐ Yes ☐ No

**If yes:**

3. Did the patient die pre-hospital?
   - ☐ Yes
   - ☐ No

   3.1 If yes, did the patient die during transport?
   - ☐ Yes (please specify with which organisation)

     ________________________________

   - ☐ No

4. Did the patient die in-hospital?
   - ☐ Yes
   - ☐ No

   4.1 If yes, in which location did the patient die?
   - ☐ ED
   - ☐ Radiology
   - ☐ Angiography
   - ☐ OT
   - ☐ ICU
   - ☐ HDU
   - ☐ Ward
   - ☐ Other (please specify)

     ________________________________

5. Was a toxicology screen conducted?
   - ☐ Yes
   - ☐ No

6. Was a post mortem completed?
   - ☐ Yes
   - ☐ No

   6.1 If yes, what type of post mortem was completed?
   - ☐ Open
   - ☐ Closed

   6.2 Is the report available?
   - ☐ Yes
   - ☐ No

2. Categorise problem:
   - ☐ Clinical
     - ○ Airway (please explain)

       ________________________________

     - ○ Breathing (please explain)

       ________________________________

     - ○ Circulation (please explain)

       ________________________________

     - ○ Disability (please explain)

       ________________________________

     - ○ Exposure/temperature (please explain)

       ________________________________

   - ☐ Systems
   - ☐ Communication
   - ☐ Unable to categorise
   - ☐ No problem identified

Comments:

# 5. Specific services involved in the care delivery problem

Indicate which department and staff were involved in the care delivery problem.

| Department | Agency/site/specialty level of staff (eg. staff specialist, P1 paramedic) |
|---|---|
| ☐ Angiography | Click here to enter text. |
| ☐ Cardiothoracic surgery | Click here to enter text. |
| ☐ Emergency Department | Click here to enter text. |
| ☐ General Surgical<br>☐ Paediatric<br>☐ Non-paediatric | Click here to enter text. |
| ☐ ICU/ HDU | Click here to enter text. |
| ☐ Mental health service | Click here to enter text. |
| ☐ Neurosurgery | Click here to enter text. |
| ☐ Operating theatre | Click here to enter text. |
| ☐ Orthopaedic | Click here to enter text. |
| ☐ Out of hospital service<br>☐ Fixed wing<br>☐ Helicopter<br>☐ Road | Click here to enter text. |
| ☐ Pathology | Click here to enter text. |
| ☐ Plastic surgery | Click here to enter text. |
| ☐ Radiology | Click here to enter text. |
| ☐ Rehabilitation | Click here to enter text. |
| ☐ Urology | Click here to enter text. |
| ☐ Other (please specify)<br>_______________________________ | Click here to enter text. |

# 6. Factors contributing to the care delivery problem

This section can be populated from information from the medical records or from the staff interviews.

| | | | | |
|---|---|---|---|---|
| **Equipment** | **1. Did equipment contribute to difficulties in delivering the required care?**<br>☐ Yes … please complete question 1.1 to 1.6 below<br>☐ No … please proceed to question 2<br>☐ Not known … please proceed to question 2 | **Yes** | **No** | **NA** |
| | 1.1. Lack of medical equipment | ☐ | ☐ | ☐ |
| | 1.2. Medical equipment breakage or failure | ☐ | ☐ | ☐ |
| | 1.3. Equipment failure – design | ☐ | ☐ | ☐ |
| | 1.4. Medical equipment not elsewhere classified | ☐ | ☐ | ☐ |
| | 1.5. Non-medical equipment | ☐ | ☐ | ☐ |
| | 1.6. Medical supplies | ☐ | ☐ | ☐ |
| | Provide further detail (if necessary) on any error identified in relation to this section:<br>Click here to enter text. | | | |

| | | | | |
|---|---|---|---|---|
| **Work environment** | **2. Did the work environment contribute to difficulties in delivering the required care?**<br>☐ Yes … please complete question 2.1 to 2.6 below<br>☐ No … please proceed to question 3<br>☐ Not known … please proceed to question 3 | **Yes** | **No** | **NA** |
| | 2.1. Light | | | |
| | 2.1.1. No or too little light | ☐ | ☐ | ☐ |
| | 2.1.2. Too much light | ☐ | ☐ | ☐ |
| | 2.1.3. Light not elsewhere classified | ☐ | ☐ | ☐ |
| | 2.2. Temperature | | | |
| | 2.2.1. Too hot | ☐ | ☐ | ☐ |
| | 2.2.2. Too cold | ☐ | ☐ | ☐ |
| | 2.2.3. Temperature not elsewhere classified | ☐ | ☐ | ☐ |
| | 2.3. Noise | | | |
| | 2.3.1. Too noisy | ☐ | ☐ | ☐ |
| | 2.3.2. Too quiet | ☐ | ☐ | ☐ |
| | 2.3.3. Noise not elsewhere classified | ☐ | ☐ | ☐ |
| | 2.4. Physical layout | | | |
| | 2.4.1. Isolation | ☐ | ☐ | ☐ |
| | 2.4.2. Poor access | ☐ | ☐ | ☐ |
| | 2.4.3. Physical layout not elsewhere classified | ☐ | ☐ | ☐ |
| | 2.5. Security | ☐ | ☐ | ☐ |
| | 2.6. Work environment not elsewhere classified (please specify)<br>Click here to enter text. | ☐ | ☐ | ☐ |
| | Provide further detail (if necessary) on any error identified in relation to this section:<br>Click here to enter text. | | | |

| | | Yes | No | NA |
|---|---|---|---|---|
| Staff action | **3. Did staff action contribute to difficulties in delivering the required care?**<br>☐ Yes … please complete question 3.1 to 3.7 below<br>☐ No … please proceed to question 4<br>☐ Not known … please proceed to question 4 | | | |
| | 3.1. Verbal communication and written documentation issues (includes misinterpretation, misunderstanding and inappropriate communication with the patient) | | | |
| | 3.1.1. Verbal communication with patient (please describe)<br>Click here to enter text. | ☐ | ☐ | ☐ |
| | 3.1.2. Verbal communication with staff – handover (please describe)<br>Click here to enter text. | ☐ | ☐ | ☐ |
| | 3.1.3. Verbal communication with staff – not during handover (please describe)<br>Click here to enter text. | ☐ | ☐ | ☐ |
| | 3.1.4. Written communication with patient (please describe)<br>Click here to enter text. | ☐ | ☐ | ☐ |
| | 3.1.5. Written communication with staff (please describe)<br>Click here to enter text. | ☐ | ☐ | ☐ |
| | 3.1.6. Other communication issue (please describe)<br>Click here to enter text. | ☐ | ☐ | ☐ |
| | 3.2. Medical task failure<br>(Execution failures in medical treatment, such as while performing surgical tasks, or task performance related to medical treatment, such as diagnostic tests. It also includes not checking medical equipment is correct and/or functioning correctly prior to use) | | | |
| | 3.2.1. Skill-based error | ☐ | ☐ | ☐ |
| | 3.2.2. Rule-based error | ☐ | ☐ | ☐ |
| | 3.2.3. Knowledge-based error | ☐ | ☐ | ☐ |
| | 3.2.4. Violation | ☐ | ☐ | ☐ |
| | 3.2.9. Not known | ☐ | ☐ | ☐ |
| | 3.3. Monitoring<br>(Lack or inadequate monitoring or follow-up of patient status, such as the monitoring of vital signs. This includes the monitoring of medication levels) | | | |
| | 3.3.1. Skill-based error | ☐ | ☐ | ☐ |
| | 3.3.2. Rule-based error | ☐ | ☐ | ☐ |
| | 3.3.3. Knowledge-based error | ☐ | ☐ | ☐ |
| | 3.3.4. Violation | ☐ | ☐ | ☐ |
| | 3.3.9. Not known | ☐ | ☐ | ☐ |
| | 3.4. Delay<br>(Delays in diagnosis or treatment of the patient, e.g. delays in obtaining or reviewing test results, transfer to surgical intervention or performing x-rays) | | | |
| | 3.4.1. Skill-based error | ☐ | ☐ | ☐ |
| | 3.4.2. Rule-based error | ☐ | ☐ | ☐ |
| | 3.4.3. Knowledge-based error | ☐ | ☐ | ☐ |
| | 3.4.4. Violation | ☐ | ☐ | ☐ |
| | 3.4.9. Not known | ☐ | ☐ | ☐ |

| | | Yes | No | NA |
|---|---|---|---|---|
| | 3.5. Misdiagnosis<br>(Misdiagnosis of a patient's ailment, including the severity of the ailment. Misdiagnosis refers to an incorrect or incomplete diagnosis of a health condition) | | | |
| | 3.5.1. Skill-based error | ☐ | ☐ | ☐ |
| | 3.5.2. Rule-based error | ☐ | ☐ | ☐ |
| | 3.5.3. Knowledge-based error | ☐ | ☐ | ☐ |
| | 3.5.4. Violation | ☐ | ☐ | ☐ |
| | 3.5.9. Not known | ☐ | ☐ | ☐ |
| | 3.6. Medication issue<br>(Medication errors, including prescribing, transcribing, dispensing, or administering medication. Also includes computer entry error in relation to medication (e.g. incorrect data are entered into a computer system regarding medication type, dose or route). This specifically does not include issues related to documentation or monitoring of medications) | | | |
| | 3.6.1. Skill-based error | ☐ | ☐ | ☐ |
| | 3.6.2. Rule-based error | ☐ | ☐ | ☐ |
| | 3.6.3. Knowledge-based error | ☐ | ☐ | ☐ |
| | 3.6.4. Violation | ☐ | ☐ | ☐ |
| | 3.6.9. Not known | ☐ | ☐ | ☐ |
| | 3.7. Human factors not elsewhere classified<br>(Other human factors not elsewhere classified) | | | |
| | 3.7.1. Skill-based error | ☐ | ☐ | ☐ |
| | 3.7.2. Rule-based error | ☐ | ☐ | ☐ |
| | 3.7.3. Knowledge-based error | ☐ | ☐ | ☐ |
| | 3.7.4. Violation | ☐ | ☐ | ☐ |
| | 3.7.9. Not known | ☐ | ☐ | ☐ |
| | Please specify: Click here to enter text. | | | |
| | Provide further detail (if necessary) on any error identified in relation to this section:<br>Click here to enter text. | | | |

| | | | | |
|---|---|---|---|---|
| **Patient** | **4. Did the patient contribute to difficulties in delivering the required care?**<br>☐ Yes ... please complete question 4.1 to 4.7 below<br>☐ No ... please proceed to question 5<br>☐ Not known ... please proceed to question 5 | | | |
| | | **Yes** | **No** | **NA** |
| | 4.1. Physical health (pre-existing) | | | |
| | 4.1.1. Pre-existing disease or physical disability | ☐ | ☐ | ☐ |
| | 4.1.2. Physical characteristic | ☐ | ☐ | ☐ |
| | 4.1.3. Intellectual disability | ☐ | ☐ | ☐ |
| | 4.1.4. Psychological disturbance | ☐ | ☐ | ☐ |
| | 4.1.5. Sensory impairment – vision | ☐ | ☐ | ☐ |
| | 4.1.6. Sensory impairment – hearing | ☐ | ☐ | ☐ |
| | 4.1.7. Physical health not elsewhere classified. Please specify:<br>Click here to enter text. | ☐ | ☐ | ☐ |

| | | Yes | No | NA |
|---|---|---|---|---|
| Patient | 4.2. Health state<br>(A change or alteration in health state, either physical or mental health state, which occurred during the current episode of care that played a part in the adverse incident) | | | |
| | 4.2.1. Airway obstruction | ☐ | ☐ | ☐ |
| | 4.2.2. Respiratory failure | ☐ | ☐ | ☐ |
| | 4.2.3. Coagulopathy | ☐ | ☐ | ☐ |
| | 4.2.4. Uncontrolled bleeding | ☐ | ☐ | ☐ |
| | 4.2.5. Deterioration in physical condition | ☐ | ☐ | ☐ |
| | 4.2.6. Severity of injury | ☐ | ☐ | ☐ |
| | 4.2.7. Infection | ☐ | ☐ | ☐ |
| | 4.2.8. Fatigue | ☐ | ☐ | ☐ |
| | 4.2.9. Health state not elsewhere classified. Please specify:<br>Click here to enter text. | ☐ | ☐ | ☐ |
| | 4.3. Communication issues<br>(Poor communication by the patient contributed to the incident. This includes language barriers, where the patient does not speak English, or when the patient either unintentionally or intentionally does not disclose information to medical staff) | | | |
| | 4.3.1. Language barrier – child | ☐ | ☐ | ☐ |
| | 4.3.2. Language barrier – parent | ☐ | ☐ | ☐ |
| | 4.3.3. Not disclosing information – unintentional | ☐ | ☐ | ☐ |
| | 4.3.4. Not disclosing information – intentional | ☐ | ☐ | ☐ |
| | 4.4. Medication<br>(Prescription medication use by the patient contributed to the incident. This includes excessive or inappropriate use of prescribed medication and also appropriate use of medication as directed) | | | |
| | 4.4.1. Prescribed drug(s) – excessive or inappropriate use | ☐ | ☐ | ☐ |
| | 4.4.2. Prescribed drug(s) – appropriate use | ☐ | ☐ | ☐ |
| | 4.5. Toxicology<br>(Excessive or inappropriate use of alcohol or illicit drugs by the patient contributed to the incident) | | | |
| | 4.5.1. Alcohol consumption | ☐ | ☐ | ☐ |
| | 4.5.2. Non-prescribed or illicit drug consumption | ☐ | ☐ | ☐ |
| | 4.5.3. Other toxicology. Please specify:<br>Click here to enter text. | ☐ | ☐ | ☐ |
| | 4.6. Clothing<br>(Inappropriate clothing worn by the patient contributed to the incident) | | | |
| | 4.6.1. Flammable | ☐ | ☐ | ☐ |
| | 4.6.2. No helmet | ☐ | ☐ | ☐ |
| | 4.6.3. No protective wear | ☐ | ☐ | ☐ |
| | 4.7. Patient characteristics not elsewhere classified<br>(Other characteristics of the patient that contributed to the incident not elsewhere classified)<br>Please specify: Click here to enter text. | ☐ | ☐ | ☐ |
| | Provide further detail (if necessary) on any error identified in relation to this section:<br>Click here to enter text. | | | |

<table>
<tr><td rowspan="8">Organisational factors</td><td colspan="4">5. Did organisational factors contribute to difficulties in delivering the required care?<br>☐ Yes ... please complete question 5.1 to 5.5 below<br>☐ No ... please proceed to question 6<br>☐ Not known ... please proceed to question 6</td></tr>
<tr><td></td><td>Yes</td><td>No</td><td>NA</td></tr>
<tr><td>5.1. Work practices, policies or guidelines<br>5.1.1. Work practice, but no policy/guidelines<br>5.1.2. Policy/guidelines exist, but are unclear/inconsistent/inadequate<br>5.1.3. Policy/guidelines exist, but are not followed<br>5.1.4. Policy/guidelines not elsewhere classified. Please specify:<br>Click here to enter text.</td><td>☐<br>☐<br>☐<br>☐</td><td>☐<br>☐<br>☐<br>☐</td><td>☐<br>☐<br>☐<br>☐</td></tr>
<tr><td>5.2. Supervision<br>(Events resulting from poor, inadequate or an absence of supervision of a junior member of the medical team)</td><td>☐</td><td>☐</td><td>☐</td></tr>
<tr><td>5.3. Organisational resources<br>5.3.1. Staffing issues (eg. skill mix)<br>5.3.2. No bed available (at treating site)<br>5.3.3. No bed available (at referring site)<br>5.3.4. Organisational resources not elsewhere classified. Please specify: Click here to enter text.</td><td>☐<br>☐<br>☐<br>☐</td><td>☐<br>☐<br>☐<br>☐</td><td>☐<br>☐<br>☐<br>☐</td></tr>
<tr><td>5.4. Work pressure<br>(Work that was being performed under unusual time pressure or haste)</td><td>☐</td><td>☐</td><td>☐</td></tr>
<tr><td>5.5. Organisation factors not elsewhere classified<br>(Other organisational factors not elsewhere classified)<br>Please specify: Click here to enter text.</td><td>☐</td><td>☐</td><td>☐</td></tr>
<tr><td colspan="4">Provide further detail (if necessary) on any error identified in relation to this section:<br>Click here to enter text.</td></tr>
</table>

<table>
<tr><td rowspan="8">Individual factors</td><td colspan="4">6. Did individual factors contribute to difficulties in delivering the required care?<br>☐ Yes ... please complete question 6.1 to 6.5 below<br>☐ No ... please proceed to question 7<br>☐ Not known ... please proceed to question 7</td></tr>
<tr><td></td><td>Yes</td><td>No</td><td>NA</td></tr>
<tr><td>6.1. Training<br>(Poor, inadequate or a lack of training of a member of the medical team)</td><td>☐</td><td>☐</td><td>☐</td></tr>
<tr><td>6.2. Experience<br>(Lack of skill or competence to perform the task (e.g. training received, but no experience in performing the task or not skilled in performing the task))</td><td>☐</td><td>☐</td><td>☐</td></tr>
<tr><td>6.3. Fatigue<br>(Events resulting from fatigue of the staff member)</td><td>☐</td><td>☐</td><td>☐</td></tr>
<tr><td>6.4. Stress<br>(Events resulting from the staff member experiencing stress)</td><td>☐</td><td>☐</td><td>☐</td></tr>
<tr><td>6.5. Individual factors not elsewhere classified<br>(Other individual factors not elsewhere classified)<br>Please specify: Click here to enter text.</td><td>☐</td><td>☐</td><td>☐</td></tr>
<tr><td colspan="4">Provide further detail (if necessary) on any error identified in relation to this section:<br>Click here to enter text.</td></tr>
</table>

| Other factors | **7. Did any other factors contribute to difficulties in delivering the required care?**<br>☐ Yes … please specify in the text box below<br>☐ No … please proceed to Section 7. Outcome<br>☐ Not known … please proceed to Section 7. Outcome |
|---|---|
| | Please specify: Click here to enter text. |

# 7. Outcome

**Tick the option that best describes the incident:**

☐ Clinically preventable trauma death
☐ Clinically near-preventable trauma death
☐ Clinically non-preventable trauma death
☐ Near miss of death
☐ Near miss of incident that did not result in death
☐ Preventable error causing lasting disability
☐ No problems identified

| |
|---|
| Comments: |

# 8. Positives of care

What were the positive aspects of care the patient received?

| |
|---|
| Click here to enter text. |

# 9. Prior knowledge

Did you have prior knowledge of this case? ☐ Yes ☐ No

Comments:

# 10. Panel discussion

This section is to be completed after panel discussion.

**Summary of review and recommendations**
Click here to enter text.

**Interview staff involved?**

☐ Yes
☐ No

Detail: Click here to enter text.
